# Activity of putative orexin neurons during cataplexy

**DOI:** 10.1186/s13041-022-00907-w

**Published:** 2022-03-04

**Authors:** Shi Zhou, Akira Yamashita, Jingyang Su, Yang Zhang, Wuyang Wang, Liying Hao, Akihiro Yamanaka, Tomoyuki Kuwaki

**Affiliations:** 1 Department of Physiology, Kagoshima University Graduate School of Medical and Dental Sciences, Kagoshima, 890-8544 Japan; 2 Department of Pharmaceutical Toxicology, China Medical University, Shenyang, 110122 Liaoning China; 3 Jiangsu Province Key Laboratory of Anesthesiology, Xuzhou Medical University, Xuzhou, 221002 Jiangsu China; 4 Department of Neuroscience II, Research Institute of Environmental Medicine, Nagoya University, Nagoya, 464-8601 Japan

**Keywords:** Cataplexy, Narcolepsy, Fiber photometry, Orexin knockout mice, Positive emotion

## Abstract

It is unclear why orexin-deficient animals, but not wild-type mice, show cataplexy. The current hypothesis predicts simultaneous excitation of cataplexy-inhibiting orexin neurons and cataplexy-inducing amygdala neurons. To test this hypothesis, we measured the activity of putative orexin neurons in orexin-knockout mice during cataplexy episodes using fiber photometry. We created two animal models of orexin-knockout mice with a GCaMP6 fluorescent indicator expressed in putative orexin neurons. We first prepared orexin-knockout mice crossed with transgenic mice carrying a tetracycline-controlled transactivator transgene under the control of the orexin promoter. TetO-GCaMP6 was then introduced into mice via an adeno-associated virus injection or natural crossing. The resulting two models showed restricted expression of GCaMP6 in the hypothalamus, where orexin neurons should be located, and showed excitation to an intruder stress that was similar to that observed in orexin-intact mice in our previous study. The activity of these putative orexin neurons increased immediately before the onset of cataplexy-like behavior but decreased (approximately − 20% of the baseline) during the cataplexy-like episode. We propose that the activity of orexin neurons during cataplexy is moderately inhibited by an unknown mechanism. The absence of cataplexy in wild-type mice may be explained by basal or residual activity-induced orexin release, and emotional stimulus-induced counter activation of orexin neurons may not be necessary. This study will serve as a basis for better treatment of cataplexy in narcolepsy patients.

## Introduction

Cataplexy is an emotionally triggered loss of muscle strength and postural collapse threatening daily life of narcolepsy patients. Cataplexy is a major symptom of narcolepsy, which is caused by abnormal loss of orexin (hypocretin)-producing neurons in humans [1], orexin-deficiency in mice [2], and orexin receptor mutation in dogs [3].

It is unclear why the absence of orexin neuronal transmission results in cataplexy. The current hypothesis holds that it involves simultaneous excitation of cataplexy-inhibiting orexin neurons and cataplexy-inducing amygdala neurons [4–7]. An increase in orexin levels in the amygdala reduces cataplexy [5], indicating a cataplexy-inhibiting role for orexin neurons. Furthermore, activation of orexin neurons results in the inhibition of the amygdala via the activation of serotonin neurons in the dorsal raphe, which receive orexinergic innervation [6, 8]. Lesion or inactivation of the amygdala in orexin-deficient mice reduced cataplexy [4, 6], indicating a cataplexy-inducing role for amygdala neurons. A microdialysis study showed orexin spillover with positive emotions in the amygdala of human patients with resistant epilepsy (but not narcolepsy) [9]. Although these observations fit the above hypothesis well, excitation of orexinergic neurons during cataplexy episodes in a time resolution of seconds has never been observed.

In addition to the awake-stabilizing role of orexin, orexin neuronal activity is closely linked to sympathetic autonomic outflow [10, 11]. Therefore, changes in the heart rate during cataplexy may be a good indicator of the activity of orexin neurons. In this context, a decrease in heart rate during cataplexy has been reported in narcolepsy patients [12] and narcolepsy dogs [13], indicating the inhibition of orexin neuronal activity during cataplexy, which is in contrast to the abovementioned counterbalance activation hypothesis.

We recently succeeded in assessing real-time orexin neuronal activity along with electrocardiograms in freely behaving mice using a fiber photometry system [11]. Using this system, we reported that aversive stimulus-evoked activation of orexin neurons preceded a heart rate increase. As an extension of the system, we hypothesized that it would be possible to assess putative orexin neuronal activity in orexin-knockout mice during cataplexy. Therefore, in this study, we measured putative orexin neuronal activity in two orexin-knockout mice models during cataplexy given that both activation and inhibition can be predicted from available circumstantial evidence.

## Results

### Validation of the model animals

**Fig. 1:**
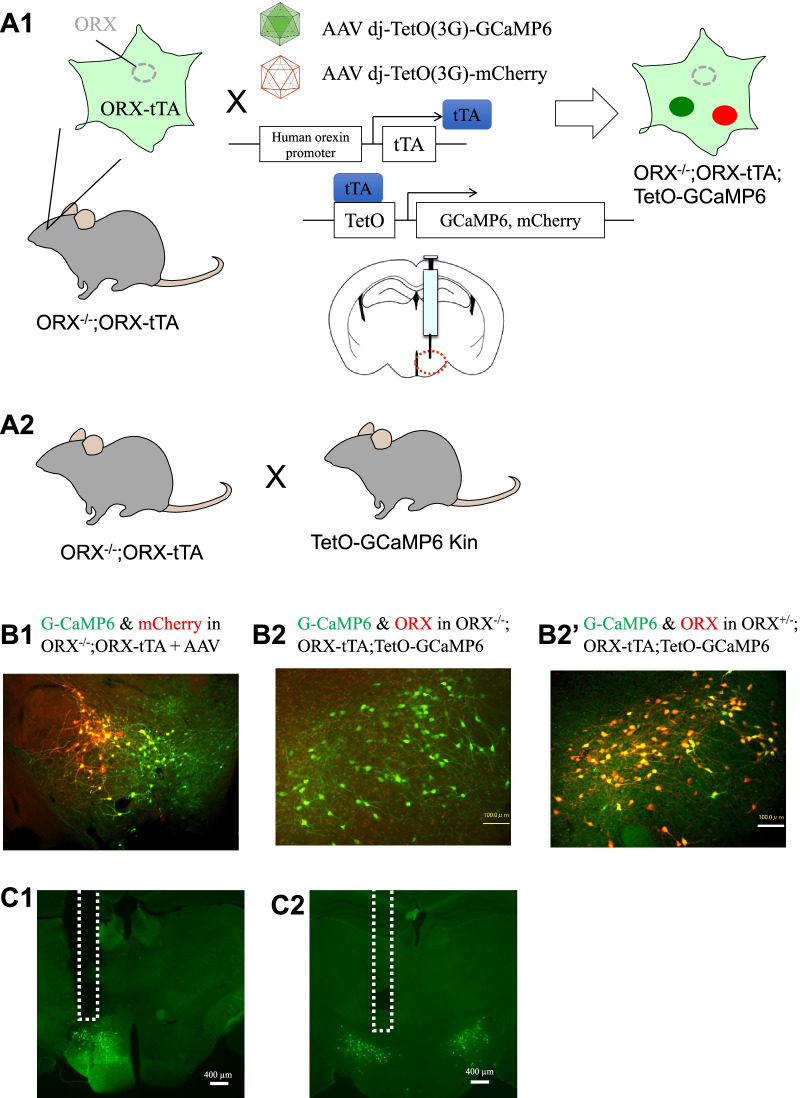
Animal models for measuring putative orexin neuronal activity. **A1** Schematic drawing showing specific expression of GCaMP6 and mCherry in putative orexin neurons by injecting adeno-associated virus (AAV) vectors into the hypothalamus of preproorexin knockout (ORX^−/−^) and orexin-tTA transgenic (ORX-tTA) double-mutant mice. We refer to this animal as “model 1” in this study. **A2** Schematic drawing showing specific expression of GCaMP6 in putative orexin neurons by crossing double mutants of ORX^−/−^ and ORX-tTA with TetO-GCaMP knock-in mice. We call this animal “model 2” in this study. **B1** Histological confirmation showing overlapping distribution of GCaMP6 (green) and mCherry (red) in the hypothalamus of a model 1 mouse. **B2** Histological confirmation showing GCaMP6 (green) in the hypothalamus of a model 2 mouse. Note that no orexin-like immunoreactivity was found, as expected. **B2′** Histological confirmation showing overlapping distribution of GCaMP6 (green) and orexin-like immunoreactivity (red) in the hypothalamus of a mouse carrying the orexin-knockout heterozygous allele in model 2. **C1** & **C2** Confirmation of fiber tracts after fluorescent recordings. The dashed line indicates the location of the inserted optic fibers. The left panel **C1** shows a brain from a model 1 mouse and the right panel **C2** shows a brain from a model 2 mouse

We used six model 1 mice (Fig. 1A1: ORX^−/−^; ORX-tTA mice injected with AAV-GCaMP6 and AAV-mCherry) and four model 2 mice (Fig. 1A2: ORX^−/−^; ORX-tTA;TetO-GCaMP6). However, we excluded the data from three animals (two model 1 and one model 2) because of failure in adeno-associated virus (AAV) injection or optic fiber implantation was noted on histological examination. In valid cases, model 1 animals showed almost exclusive expression of GCaMP6 and mCherry in the hypothalamus, where orexin neurons should be located, and the area under the successfully implanted optic fiber (Fig. 1B1; double in GCaMP6 = 74.9 ± 2.1%, double in mCherry = 91.4 ± 1.5%, mean ± SEM, n = 4). In model 2 animals, there were 87.3 ± 7.3 (mean ± SEM, n = 3) GCaMP6-positive cells per side in a slice. This number was slightly larger than that in model 1 (72.5 ± 5.1, n = 4), but the difference was not statistically significant (p = 0.176, Student’s *t*-test). We further confirmed that GCaMP6-positive cells in model 2 mice were putative orexin neurons, showing almost exclusive expression of GCaMP6 in orexin-immunoreactive cells, with more than 90% of these cells expressing GCaMP6 in ORX^±^; ORX-tTA;TetO-GCaMP6 mouse (Fig. 1B2′, n = 1). Thus, the fluorescence data described below were considered to originate from putative orexin neurons from a histological point of view.

**Fig. 2:**
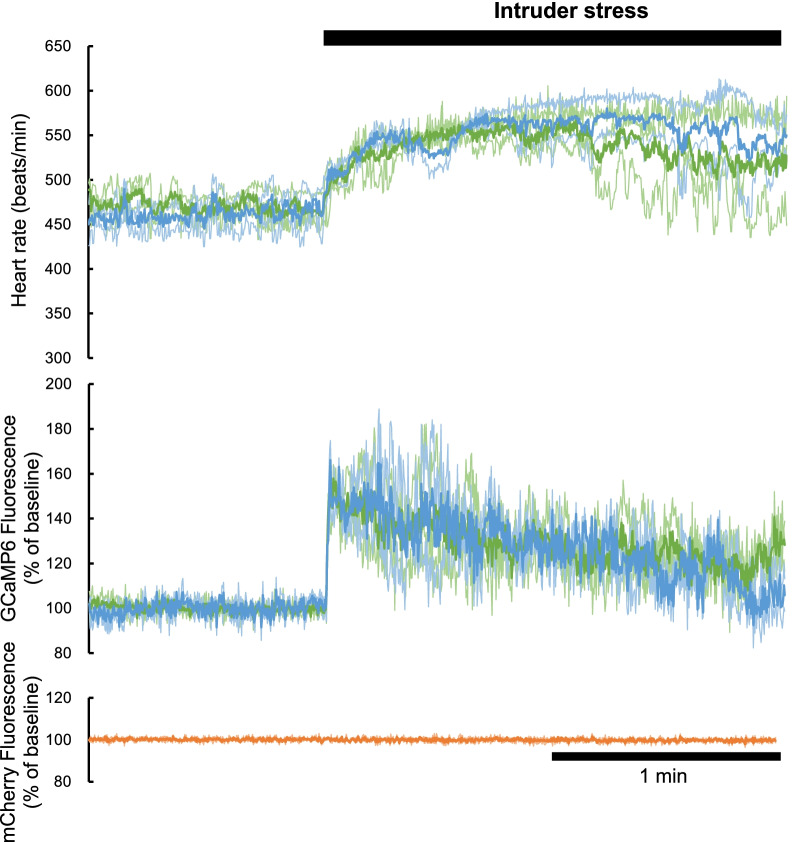
Physiological confirmation showing activation of putative orexin neurons to intruder stress. The time course of the heart rate, GCaMP6 fluorescence, and mCherry fluorescence during intruder stress are shown from the top to the bottom. We used three model 1 animals, shown in green, and two model 2 animals, shown in blue. Thin lines indicate the data from individual animals, and thick lines indicate the averaged values. Note that the averaged responses in models 1 and 2 were almost similar for both heart rate and GCaMP fluorescence. mCherry data were only obtained in three model 1 animals and showed no change on exposure to the intruder

To confirm that our data came from putative orexin neurons with physiological meanings, we recorded GCaMP6 and mCherry fluorescent signals together with ECG when the animals were confronted with intruder stress (Fig. 2). On exposure to intruder stress, GCaMP6 signals during the first 1 min increased significantly from 100.0 ± 0.02% to 135.4 ± 5.5% (n = 3, p = 0.017, two-way ANOVA followed by Sidak’s multiple comparisons test) in model 1 animals and from 99.9 ± 0.11% to 136.7 ± 7.4% (n = 2, p = 0.027) in model 2 animals. There was no statistically significant difference between the models (F_1,3_ = 0.0214, P = 0.893). In a similar manner, the heart rate increased from 471.2 ± 3.9 bpm to 539.6 ± 5.4 bpm (p = 0.002) in model 1 animals and from 461.2 ± 15.5 bpm to 544.5 ± 4.0 bpm (p = 0.002) in model 2 animals. There was no statistically significant difference between the models (F_1,3_ = 0.075, p = 0.802). In contrast, the mCherry signal (measured only in model 1 animals) did not change (100.1 ± 0.07% vs. 100.0 ± 0.14%, n = 3, P = 0.498). Thus, the fluorescence sources in models 1 and 2 were similar. More importantly, these values were similar to those obtained in orexin-preserved mice (ORX^+/+^; ORX-tTA mice injected with AAV-GCaMP6 and AAV-mCherry) in our previous study [11] (∆GCaMP ca. 40%, ∆mCherry ca. 0%). Thus, the fluorescence data shown below were considered to originate from putative orexin neurons from both histological and physiological points of view.

### GCaMP fluorescence and heart rate increases immediately before and decreases during cataplexy-like behavior

**Fig. 3:**
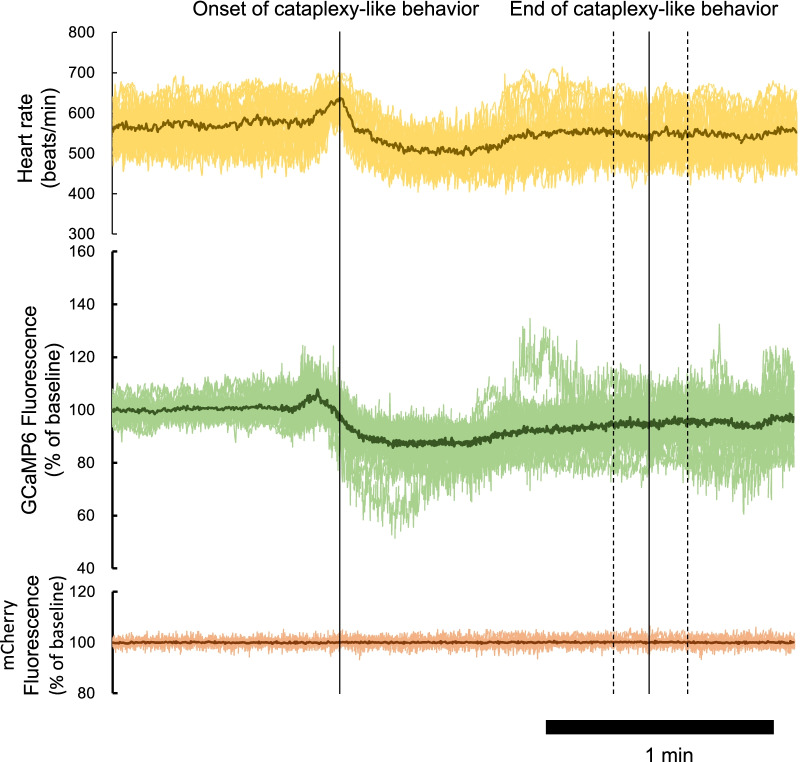
Typical tracing of the heart rate, GCaMP fluorescence, and mCherry fluorescence in a model 1 animal. The time course of the heart rate, GCaMP6 fluorescence, and mCherry fluorescence during cataplexy-like behavior are shown from the top to the bottom. During the 10-h observation period, 25 episodes of cataplexy-like behaviors were observed in this animal. Each record was aligned so that the onset time of cataplexy-like behavior was equal and is shown as overlapping thin lines. The average value from the 25 episodes is shown as a thick line. The cataplexy-like episode lasted 79.8 ± 9.7 s (mean ± SEM, range 27‒209 s, dashed lines indicate the standard error of the mean)

Cataplexy-like behavior was observed 31.8 ± 2.9 times in model 1 animals (n = 4) and 27.9 ± 3.0 times in the model 2 animals (n = 3) during a 10-h observation period. There was no statistically significant difference between the models (p = 0.405). Traces of heart rate and fluorescence were aligned so that the onset time of cataplexy-like behavior was equal and the averaged values of the time were calculated for all cataplexy-like behaviors in a single animal (Fig. 3). There was a significant increase in heart rate (from 567.7 ± 5.3 bpm to 623.9 ± 5.3 bpm, p < 0.0001, n = 25) around the onset of cataplexy-like behavior and a significant decrease (to 512.0 ± 4.7 bpm, p < 0.0001) during the cataplexy-like episode. In a similar manner, GCaMP6 fluorescence increased (from 100.0 ± 0.1% to 113.2 ± 1.2%, P < 0.0001, n = 25) almost immediately before the heart rate peak and then decreased (to 89.0 ± 1.1%, p < 0.0001) during cataplexy-like behavior. The GCaMP6 fluorescence peak (10.01 ± 1.33 s before the onset of cataplexy-like behavior) significantly preceded the heart rate peak (1.50 ± 0.47 s, p < 0.0001, n = 25, paired t-test). In contrast, mCherry fluorescence did not show any change. There was no apparent difference in the time course of changes in heart rate and GCaMP6 fluorescence among individual cataplexy-like episodes, except that they varied relatively widely during the latter part of the recordings, as shown in Fig. 3, presumably because the duration of cataplexy-like episodes varied from episode to episode (27–209 s in the animal in Fig. 3).

**Fig. 4:**
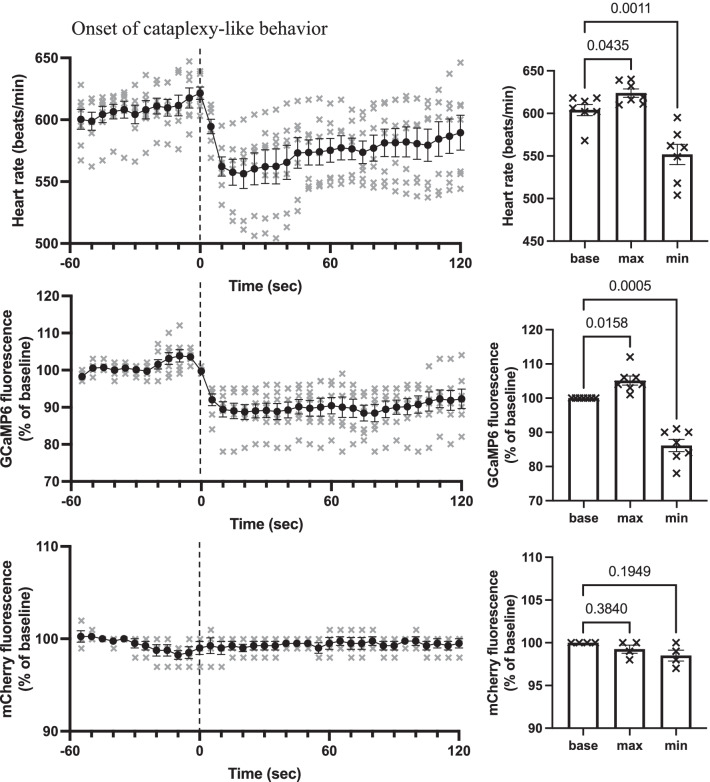
Time-related changes in the heart rate, GCaMP fluorescence, and mCherry fluorescence grouped from seven animals. Left column: Time course of the heart rate, GCaMP6 fluorescence, and mCherry fluorescence during cataplexy-like behavior are shown from the top to the bottom. The averaged value in one animal for all of the cataplexy-like episodes (see thick line in Fig. 3) was resampled in 5-s bins and was averaged for seven animals. Circles denote mean values, and vertical lines denote the standard error of the mean. The individual values are indicated by crosses. Heart rate and GCaMP fluorescence data were obtained from four model 1 animals and three model 2 animals. mCherry data were obtained from four model 1 animals. Right column: Baseline value during the 30–60 s before, maximum value during the 0–30 s before, and minimum value during the 0–90 s after the onset of cataplexy were compared. There were significant differences in heart rate values (F_2,6_ = 37.26, p = 0.0001) and GCaMP6 values (F_2,6_ = 47.75, p = 0.0001), but not in mCherry values (F_2,3_ = 4.765, p = 0.1055). P values in the figure were obtained using 1-way analysis of variance, followed by Sidak’s multiple comparison test

Using the averaged data for all cataplexy episodes in one animal (see Fig. 3) as representative data for that animal, we constructed grouped data (n = 7 animals) during cataplexy-like episodes (Fig. 4). The baseline value was defined as the average during a 30-s period from 60 s before to 30 s before the onset of cataplexy behavior. The maximum and minimum data were defined as the average of 5 s around the peak. Grouped data again showed a significant increase (Fig. 4, right panel) in heart rate around the onset of cataplexy-like behavior and a significant decrease during the cataplexy-like episode. GCaMP6 fluorescence increased almost immediately (ca. 10 s) before the onset of cataplexy-like behavior and the maximum peak of the heart rate, and then decreased during cataplexy-like behavior. Cataplexy-like behavior lasted for 87.7 ± 5.1 s (average of 7 animals), and GCaMP fluorescence returned to baseline 154.6 ± 8.5 s, about 70 s after the recovery from cataplexy. During cataplexy-like episodes, GCaMP6 fluorescence did not reach zero, indicating the existence of some residual activity in putative orexin neurons.

## Discussion

In this study, we described the transient excitation of putative orexin neurons immediately before the onset of cataplexy-like behavior and successive inhibition during the episode. These changes were followed by changes in heart rate.

### Validation of the model animals

We used two strategies to express GCaMP6 in putative orexin neurons in orexin-knockout mice: one involved the use of an AAV vector and the other involved natural crossing. The resulting mouse lines showed close resemblance to each other from histological (location in the lateral and dorsal hypothalamic area) and physiological (response to intruder stress) points of view, indicating reasonable validity of the models. Most importantly, the changes in fluorescence of GCaMP6 and mCherry were similar to those obtained in orexin-preserved mice (ORX^+/+^; ORX-tTA mice injected with AAV-GCaMP6 and AAV-mCherry) in our previous study [11] (∆GCaMP approximately 40%, ∆mCherry approximately 0%), indicating that intruder stress activated both intact orexin neurons and putative orexin neurons in a similar manner.

Changes in heart rate in the current study (ca. 70 bpm) were relatively smaller than in those reported in a previous study [11] (ca. 150 bpm). This result indicated that orexin neurotransmitters from orexin neurons contributed substantially to the increase in heart rate [14] and that an orexin neuron-independent pathway, such as that observed in glutamatergic neurons in the dorsomedial hypothalamus [15], also helped increase the heart rate in response to intruder stress.

During cataplexy-like behavior in this study, we found a marked decrease in heart rate (Fig. 4), which closely resembles that reported in narcolepsy patients [12] and narcolepsy dogs [13]. Therefore, we considered that the cataplexy episode could be well-estimated by the current method of behavioral observation, without assessment of the EEG. In dogs with narcolepsy [13] but not in human patients [12], an increase in heart rate at around the onset of a cataplexy attack has also been reported. Our mouse models closely resembled the dog cases. Differences in the deficit of orexin-signaling system (mice: deficiency of orexin peptide [2], dog: non-functional orexin receptor [3], human: ablation of orexin-synthesizing neurons [1]) may be related to species differences in the heart rate response because ablation of orexin-synthesizing neurons extinguishes not only orexin but also coexisting neurotransmitters/modulators expressed by the neurons [16, 17]. It may be interesting to note here that a functional magnetic resonance imaging study in narcolepsy patients showed marked reductions in hypothalamic activity during cataplexy attack [18], although the relevant neuronal identity was unclear.

### Inhibition of the putative orexin neurons during cataplexy

Excitation of putative orexin neurons, as shown by an increase in GCaMP6 fluorescence (Figs. 3, 4), may fit the counterbalance hypothesis of simultaneous activation of cataplexy-promoting amygdala neurons and cataplexy-inhibiting ORX neurons [4–7]. In this case, however, it seemed difficult to explain how short-lasting (ca. 15 s) excitation before the onset of cataplexy counteracts subsequent relatively long-lasting (30–200 s) cataplexy episodes. A short-lasting excitation may release ORX in the wild-type mice and amplify ORX neuronal activity through orexin-2 receptor expressed in the ORX neurons [19]. However, excitation of ORX neurons by orexins lasts for no more than 30 s, at least in vitro [19]. We do not know whether such excitation, if any, might be enough to overcome succeeding inhibitory inputs to the orexin neurons. The second interpretation of the current results may be that the reduction of heart rate and GCaMP6 signals may indicate a transient slow-down of hyper arousal state with high sympathetic tones, because cataplexy often occurs in the middle of highly active behavior in ORX-deficient mice [2, 20, 21]. The lowered signals, especially the heart rate (~ 550 bpm, Fig. 4), may show even higher level than those seen during the quiet wake period (~ 470 bpm, before intruder stress in Fig. 2); thus, these reductions may simply reflect the end of hyper activity of ORX knockout mice before a cataplexy episode. If this interpretation was true, then putative ORX neurons in ORX knockout mice seemed to not receive counter activation, at least during the cataplexy period. The third interpretation of the current results may be that cataplexy-inhibiting orexin neurons are subpopulations of ORX neurons, and even when these neurons are excited during cataplexy, inhibition of other subpopulations of ORX neurons mask such excitation. Since orexin neurons play multiple roles such as wake-promotion, food-seeking, motivation, and sympathetic activation [22, 23], some researchers have proposed the existence of subpopulations [24]. However, even if there are subpopulations of ORX neurons, the possible relationship between the activity of the subpopulation and cataplexy attack is highly speculative at present. The fourth and most simple explanation is that low activity of ORX neurons, if not zero, is sufficient to suppress cataplexy in wild-type mice. This explanation is supported by the observation that partial (< 90%) destruction of ORX neurons did not result in cataplexy [25]. Partial inhibition of putative ORX neuronal activity in this study indicated partial inhibition, but not excitation, of ORX neuronal activity in the wild-type mice in a situation where cataplexy would occur in the ORX knockout mice. Our notion is supported by evidence that partial blockade of orexin receptors by hypnotic drugs did not induce cataplexy [26] and that ectopic supplementation of ORX is effective in reducing cataplexy in ORX neuron-ablated mice [27].

### Limitations

At this time, we do not know how and why orexin neurons receive inhibitory input during cataplexy. Orexin neurons express opioid receptors, and activation of the receptor inhibits orexin neuronal activity at least in vitro [28]. Positive emotions induce both cataplexy [21, 29] and opioid release [30]. Sympathetic activation, a role of orexin neurons [31], is not as strong during negative emotions as during positive emotions [32]. These factors may underlie the mechanism, but the biological meaning of this inhibition during cataplexy requires further study.

## Conclusions

Here, we described the transient excitation of putative orexin neurons immediately before the onset of cataplexy-like behavior and their subsequent inhibition during the cataplexy-like episode. These results were not in good agreement with the counterbalance hypothesis. We propose a simpler hypothesis as follows. The absence of cataplexy in wild-type mice may be explained by basal or residual activity-induced orexin release, and emotional stimulus-induced counter activation of orexin neurons may not be necessary. This study will serve as a basis for better treatment of cataplexy in narcolepsy patients.

## Materials and methods

### Ethics approval

All experiments were conducted at Kagoshima University according to the guiding principles for the care and use of animals in the field of physiological sciences, published by the Physiological Society of Japan (2015), and were approved by the Experimental Animal Research Committee of Kagoshima University (MD17105 and MD20004).

### Animals

We used two genetically engineered animal models of narcolepsy, which express the calcium indicator GCaMP6 in their putative orexin neurons. First, prepro-orexin-knockout mice [2] (ORX^−/−^) were crossed with transgenic mice carrying a tetracycline-controlled transactivator transgene (tTA) under the control of the orexin promoter [11] (ORX-tTA). The resulting ORX^−/−^; ORX-tTA mice were expected to express tTA, but not orexin, in their “orexin” neurons (Fig. 1A1). By injecting AAV-GCaMP6 (*stereotaxic injection of AAV* section) into the hypothalamus of these mice, we obtained ORX^−/−^; ORX-GCaMP6 (model 1) mice. The second model was constructed by crossing ORX^−/−^; ORX-tTA mice with TetO-GCaMP6 mice (Fig. 1A2). In TetO-GCaMP6 knock-in mice, the beta-actin gene was modified to convey a gene encoding tetracycline operator (TetO)-GCaMP6 [33] (B6; 129-Actb < tm3.1(tetO-GCaMP6)*Kftnk* > , obtained from RIKEN Bio Resource Research Center, RBRC09552). We used these two models to examine whether these two models, which are conceptually the same but technically different, would yield similar results.

All mice were housed in a room maintained at 22–24 °C with lights on at 19:00 and off at 7:00 for at least 2 weeks before experimentation began. We selected a reversed light/dark cycle so that the experimenters could observe mice in their active nocturnal phase of behavior during the daytime. We used male mice to avoid possible effects from an estrous cycle, as female hormonal cycles, at least in humans, may have some effect on sleep-related disorders, including narcolepsy with cataplexy [34]. We housed mice individually from the start of the reversed light/dark cycle to avoid any possible social rank effect on male behavior [35].

### Stereotaxic injection of AAV

Under isoflurane anesthesia (2%, inhalation) using a stereotaxic instrument (ST-7, Narishige, Tokyo, Japan), a viral mixture consisting of recombinant AAV-tetO(3G)-G-CaMP6 (serotype: DJ; 600 nl/injection, 3 × 10^12^ copies/ml) and AAV-tetO(3G)-mCherry (serotype: DJ; 600 nl/injection, 6 × 10^12^ copies/ml) was stereotaxically injected into the left side of the hypothalamic perifornical area in ORX^−/−^;ORX-tTA mice (Fig. 1A1). All AAVs used in this study were produced by Yamanaka’s Laboratory at Nagoya University, Japan [11]. The injection site was as follows: 1.5-mm posterior from the bregma, 0.8-mm lateral, and 5.0-mm ventral from the dura.

### In vivo recordings of neuronal activity using a fiber photometry system and cardiovascular parameters using a radio-telemetry system

We used our previously reported method [11]. In brief, 2–3 weeks after viral injection (model 1) or 1 week before the measurement (model 2), mice were anesthetized with 2% isoflurane and were surgically implanted with a guide cannula (diameter: 600 µm, length of guide: 8 mm, made by LUCIR, Tsukuba, Japan) to place the optical fiber immediately above the hypothalamus (1.5 mm posterior to the bregma, 0.8 mm lateral, 4.0 mm ventral) to record orexin neuronal activity. Immediately after performing the guide cannula implantation, we performed additional surgery to implant a radio-telemetry transducer (TA11PA-C20, Data Sciences International, St. Paul, MN, USA) into the abdominal cavity of the mice to record electrocardiograms (lead II). During all surgeries, care was taken to maintain body temperature. After surgery, the mice were treated with penicillin, an analgesic, and buprenorphine. For recovery, mice were individually housed and monitored and had access to food and water ad libitum for at least 1 week.

A fiber photometry system (COME2-FTR/OPT, LUCIR, Tsukuba, Japan) was used to record the activity of orexin neurons in freely moving mice, as previously described [11]. During the measurement, a dummy fiber was removed and a 400-μm silica fiber (LUCIR) was inserted through the guide cannula into the brain. G-CaMP6 fluorescence and mCherry fluorescence were collected using the same silica fiber. The respective G-CaMP6 or mCherry fluorescence was guided to individual photomultipliers. The signal was digitized at 100 Hz using a data acquisition system (PowerLab16/35, ADInstruments, New South Wales, Australia) and was recorded using LabChart software version 8 (ADInstruments Inc., Bella Vista, NSW, Australia).

On the experimental day, mice were individually placed into a recording chamber in a soundproof box with a 12-h:12-h reversed light/dark cycle for 10 h, from 8:00 (lights off at 7:00) to 18:00. The chamber was illuminated with a far infrared lamp (940 nm, SA2-IR, World Musen, Hong Kong), and a piece of chocolate was placed in the chamber at the start of observation to increase the episodes of cataplexy [21]. Mouse behavior was continuously recorded with a video camera (CBK21AF04, The Imaging Source Asia, Taipei, Taiwan) and was monitored on a personal computer located outside the soundproof box using the video capture function in LabChart. Electrocardiograms of mice were sampled through a radio-frequency receiver (RLA1020, Data Sciences International), digitally converted at 1000 Hz, and transferred to a computer with PowerLab (ADInstruments). The heart rate was calculated using the cyclic variable function in LabChart. The video movie, G-CaMP6 and mCherry fluorescence signals, electrocardiogram, and calculated heart rate were stored on a personal computer. The fluorescence signal intensity at its nadir during the 10-h observation period was defined as 0%. At this moment, the mouse seemed to be in a sleeping/rest state, as judged through video recordings. To exclude individual differences in fluorescence signal intensity, the average value from − 60 to − 30 s from the onset of cataplexy was defined as 100%.

### Stress stimulation

To test whether the putative orexin neuron in this study responded to an aversive stimulus, as did the orexin-producing neurons, we used an intruder stress test, as in our previous study [11]. This stressor was applied by placing an age-matched wild-type mouse (intruder mouse) in a small polypropylene cage into the experimental cage for 2 min. The polypropylene cage was constructed such that the intruder and resident (experimental) mice were unable to contact each other physically, but visual, auditory, and olfactory communications were possible.

### Behavioral observation of cataplexy

Cataplexy was determined according to the established criteria for mice [20], which are defined by several observable features. The first feature is an abrupt episode of nuchal atonia lasting at least 10 s. Atonia was determined to occur when mice were in a prone position with their head and belly down in the bedding with their limbs and tail typically situated straight out from the trunk. This posture shows a clear contrast to a normal sleeping position in which mice are curled up and fold their limbs and tail underneath their trunk. Furthermore, in atonia, the mouse was immobile besides the movements associated with breathing during an episode. Finally, there had to be at least 40 s of active wakefulness (moving) preceding the atonia episode. The original criteria recommended recordings of EEG, but we did not adopt EEG to avoid heavy attachments to the head with the photometry fiber and EEG cables. Therefore, we refer to “cataplexy-like behavior” instead of “cataplexy” in this manuscript.

### Immunohistochemistry

Mice were deeply anesthetized with urethane (2.0 g/kg, i.p.) and were transcardially perfused with Ringer’s solution (containing CaCl_2_), followed by 4% paraformaldehyde in 0.1 M Tris (pH 7.4) + 3 mM CaCl_2_. We added calcium to the ordinal washing and fixative solutions because in our preliminary experiment, we found that the fluorescence of GCaMP6 was better preserved with calcium supplementation. The brain was removed, post-fixed in 4% paraformaldehyde + Ca solution at 4 °C overnight, and was subsequently immersed in phosphate buffered saline (PBS) at 4 °C for at least 2 days. A series of 40-μm sections were obtained using a vibratome (SuperMicroSlicer Zero1; DOSAKA EM, Kyoto, Japan). For staining, coronal brain sections were immersed in blocking buffer (1% normal horse serum and 0.3% Triton-X in PBS) and were then incubated with an anti-orexin A rabbit antibody (1:1000, 14346v, Peptide Institute, Osaka, Japan) at room temperature (about 20 °C) for 1 h. The sections were washed with PBS and were incubated in a CF568-conjugated anti-rabbit IgG antibody (1:500, catalogue #20098, Biotium, Heyward, CA, USA) for 2 h at room temperature. These brain sections were mounted on slides and were imaged using a fluorescence microscope (BZ-9000, Keyence, Osaka, Japan). For counting, we used one slice in which the optic fiber tract was most evident in the animal (Fig. 1C).

### Statistical analysis

Statistical analyses were performed using GraphPad Prism (GraphPad Software, La Jolla, CA, USA). To compare the two groups of data, we used the Student’s *t*-test. To compare three or more groups of means, we used one-way or two-way analysis of variance followed by Sidak’s multiple comparison tests depending on the data structure. Data were reported as means and standard error of the mean. Statistical significance was set at P < 0.05.

## Data Availability

Summary statistics are available in the article. The data that support the findings of this study are available from the corresponding author upon reasonable request.
